# 

**Published:** 1882-10

**Authors:** 


Vol. III.] TWO DOLLARS A YEAR. SINGLE COPIES 60 CENTS. [No. 4.

### THE JOURNAL OF COMPARATIVE MEDICINE AND SURGERY.

### A Quarterly Journal OF THE ANATOMY, PATHOLOGY, AND THERAPEUTICS OF THE LOWER ANIMALS.

NEW YORK:
W. L. HYDE & CO., PUBLISHERS, NO. 22 UNION SQUARE.
1882.

